# A therapeutic roadmap to restore the SORL1‐retromer recycling complex

**DOI:** 10.1002/alz.084839

**Published:** 2025-01-03

**Authors:** Kalpana M. Merchant

**Affiliations:** ^1^ Retromer Therapeutics, New York, NY USA

## Abstract

Genetic, cell biology and autopsied brain tissue studies indicate that deficits in the SORL1‐retromer complex play a critical role in the pathogenesis of Alzheimer’s disease (AD). SORL1 is an endosomal receptor that interacts with the retromer heterotrimer core complex consisting of VPS26‐VPS35‐VPS29. Together, SORL1‐retromer regulate endosomal recycling of several AD‐related cargos such as amyloid precursor protein. In cell and animal models, deficits in SORL1 or retromer core levels recapitulate the prototypical biomarker signature and cytopathology of AD. Thus, restoration of SORL1‐retromer recycling function offers an attractive therapeutic opportunity to slow or stop the progression of AD. This talk will detail a multi‐modality drug development strategy founded on precision neurology principles. Specifically, there will be a discussion on small molecules and gene therapy drug development programs targeting SORL1 and/or the retromer core as well as a comprehensive biomarker strategy that has been integrated into the programs to de‐risk and facilitate clinical drug development.

